# Aspects of statin prescribing in Norwegian counties with high, average and low statin consumption – an individual-level prescription database study

**DOI:** 10.1186/1472-6904-7-14

**Published:** 2007-12-05

**Authors:** Ingeborg Hartz, Solveig Sakshaug, Kari Furu, Anders Engeland, Anne Elise Eggen, Inger Njølstad, Svetlana Skurtveit

**Affiliations:** 1 Faculty of Health Studies, Hedmark University College, Kirkeveieen 47, 2418 Elverum, Norway; 2 Department of Pharmacy, University of Tromsø, Tromsø, Norway; 3 Norwegian Institute of Public Health, PO Box 4404 Nydalen, 0403 Oslo, Norway; 4 Department of Public Health and Primary Health Care, University of Bergen, Bergen, Norway; 5 Department of Community Medicine, University of Tromsø, Tromsø, Norway

## Abstract

**Background:**

A previous study has shown that variations in threshold and intensity (lipid goal attainment) of statins for primary prevention contribute to regional differences in overall consumption of statins in Norway. Our objective was to explore how differences in prevalences of use, dosing characteristics, choice of statin and continuity of therapy in individual patients adds new information to previous results.

**Methods:**

Data were retrieved from The Norwegian Prescription Database. We included individuals from counties with high, average, and low statin consumption, who had at least one statin prescription dispensed during 2004 (N = 40 143).

1-year prevalence, prescribed daily dose (PDD), statin of choice, and continuity of therapy assessed by mean number of tablets per day.

**Results:**

The high-consumption county had higher prevalence of statin use in all age groups.

Atorvastatin and simvastatin were dispensed in 79–87% of all statin users, and the proportion was significantly higher in the high-consumption county.

The estimated PDDs were higher than the DDDs, up to twice the DDD for atorvastatin. The high-consumption county had the highest PDD for simvastatin (25.9 mg) and atorvastatin (21.9 mg), and more users received tablets in the upper range of available strengths. Continuity of therapy was similar in the three counties.

**Conclusion:**

Although differences in age-distribution seems to be an important source of variation in statin consumption, it cannot account for the total variation between counties in Norway. Variations in prevalences of use, and treatment intensity in terms of PDD and choice of statin also affect the total consumption. The results in this study seems to correspond to previous findings of more frequent statin use in primary prevention, and more statin users achieving lipid goal in the highest consuming county.

## Background

**Figure 1:**
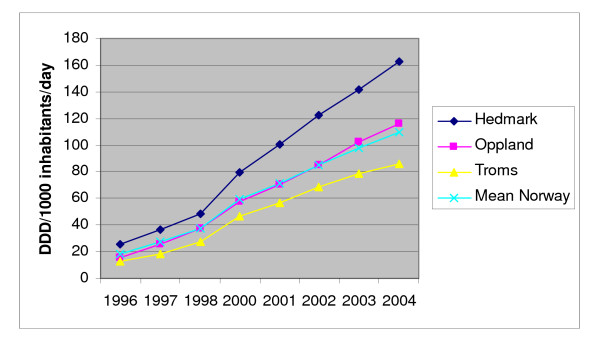
Sales of statins (ATC group C10AA) in Defined Daily Doses (DDDs) per 1000 inhabitants per day in three Norwegian counties and Norway as a whole, 1996–2004. Wholesale statistics, Norwegian Institute of Public Health [3].

During the last decade clinical data on the benefits of statins in the prevention of cardiovascular disease have accumulated [1]. Statin consumption, measured as defined daily doses (DDDs) per 1000 inhabitants per day, has increased remarkably in Norway, and is high compared with other European countries [2,3]. However, there are large and persistent regional differences in statin consumption in Norway [3]. In 2004, the top-consumption county, Hedmark, had a sales volume that was twice the level in the low-consumption county, Troms, and 40% higher than the neighbouring county, Oppland (Figure 1) [3]. Ideally, geographical variations of statin consumption should reflect variations in the size of population eligible for such therapy, as defined by the guidelines. However, according to our previous findings in a population-based study from Hedmark and Oppland, the presence of cardiovascular morbidity and risk factors were similar in the populations in the two counties [4]. Still our previous study indicated that more people received statins for primary prevention in the high-consumption county Hedmark. In addition, the statin users in the primary prevention subgroup seemed to be treated more intensively, as reflected in the higher attainment of total cholesterol (TC) targets among statin users in Hedmark [4]. The success in achieving the TC target might, however, be influenced by the use of higher dosages of statins and/or continuity of use. Based on prescription data, the main objective of the present study was to explore the following aspects of statin use in three counties with high (Hedmark), average (Oppland) and low (Troms) total statin consumption: prevalences of use, dosing characteristics, choice of statin and continuity of therapy.

## Methods

### The Norwegian Prescription Database

Data were retrieved from the Norwegian Prescription Database (NorPD), which includes prescription data from the total population (4.6 million) in Norway since 2004 [5,6]. The NorPD contains information from all prescription drugs, reimbursed or not, dispensed at pharmacies to individual patients living outside institutions. The identity of patients has been encrypted, but each record contains a unique person identifier, which makes it possible to identify all prescriptions for individuals.

We included persons from three counties with high (Hedmark), average (Oppland) and low (Troms) statin consumption; each individual had at least one prescription of a statin (ATC group C10AA) dispensed during 2004. In total, 40 143 statin users were included in our analysis: 17 954, 13 348 and 8841 from the high-, average- and low-consumption counties, respectively. The data collected were: patient's sex, age and place of residence; date of dispensing; and drug information (brand name, package size, number of packages, ATC code, DDD). Statin use in institutions (hospitals and nursing homes) accounted for less than 2% of the total statin consumption in Norway in 2004, and is not included in this analysis.

### Definitions

Period (1-year) prevalence of use was estimated by identification of individuals who had at least one statin prescription dispensed during 2004.

Distribution of all statin users according to prescribed statin substance was defined by the first prescription dispensed in 2004.

Information about the prescribed daily dose (PDD) is not yet available from the NorPD. However, statins are available in many tablet strengths [7], and validation of statins in other prescription databases have shown that the dispensed tablet strength concords highly with the PDD [8]. Based on the assumption that all users take one tablet daily, a surrogate for the PDD was calculated for all statins from the total volume dispensed in milligrams divided by the total number of dispensed tablets; this gave a mean PDD per county for all statins. In addition, the distribution of all statin users, according to tablet strength on the first prescription dispensed in 2004, was studied.

Continuity of statin use between two prescription retrievals was explored under the assumption of the use of one tablet per day as the unit. Hence, when the number of days between two prescriptions did not exceed the number of tablets dispensed by the first prescription, continuity in this period can be assumed. For every prescription followed by another prescription in the same patient, the number of tablets dispensed on the first prescription was divided by the number of days between the two prescriptions, which gave a mean daily use of tablets (tablets/day).

### Statistics

All analyses were done using SPSS 12.0 for Windows. Age and sex standardisation of total period prevalence of statin use was done by the direct method (5-year intervals) using the Norwegian population as the standard [9]. Proportions with 95% confidence intervals were used to describe statin users according to choice of statin and retrieved tablet strength in the counties. The distribution of the number of tablets/day between two prescription retrievals was skewed, and thus described by medians and interquartile ranges (IQR).

## Results

### Prevalence of statin use

**Figure 2:**
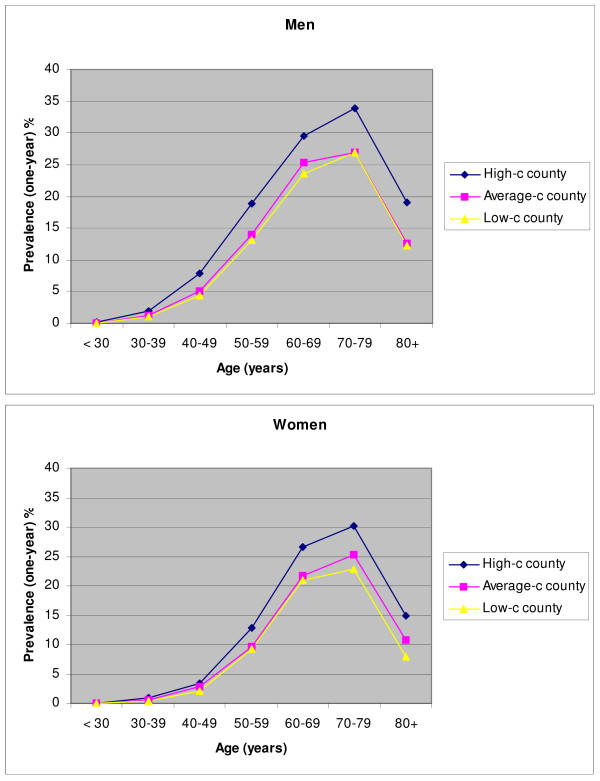
**One-year prevalence (%) of use of statins in three Norwegian counties by age and gender. Norwegian Prescription Database 2004**. High-c county: abbreviation for high-consumption county.

Figure 2 presents 1-year prevalence according to age and sex among individuals who had at least one statin dispensed during 2004. The crude prevalence (age adjusted) for men in the different counties were: 10.3% (8.9%), 7.8% (6.9%) and 6.4% (6.4%) in the high-, average- and low-consumption counties, respectively. The corresponding figures for women were: 8.8% (7.5%), 6.9% (6.0%) and 5.3% (5.4%).

The high-consumption county had higher prevalence of statin use in all age groups, even though the differences were minimised in the age adjusted prevalences. Only minor differences were observed between the two other counties. More men than women were on statins, and statin use increased by age in all three counties, peaking among 70- to 79-year-old individuals in both sexes. The prevalence of use in people aged 80 years and older was only 50% compared with the 70–79 year olds, in all counties.

### Choice of statin

**Figure 3:**
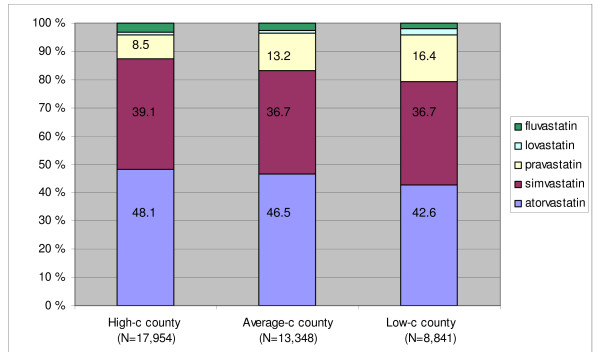
**Proportion of all users according to different statins prescribed in three Norwegian counties ^1^. Norwegian Prescription Database 2004**. High-c county: abbreviation for high-consumption county. ^1 ^Statin substance according to the first prescription dispensed in 2004.

Atorvastatin accounted for 42–47%, simvastatin for 37–40% and pravastatin for the remaining 9–17% of all statin users in the three counties (Figure 3). More users in the high-consumption county Hedmark were prescribed atorvastatin and simvastatin, whereas pravastatin constituted a larger proportion of all statin users in the two other counties.

### Prescribed daily dose

**Figure 4:**
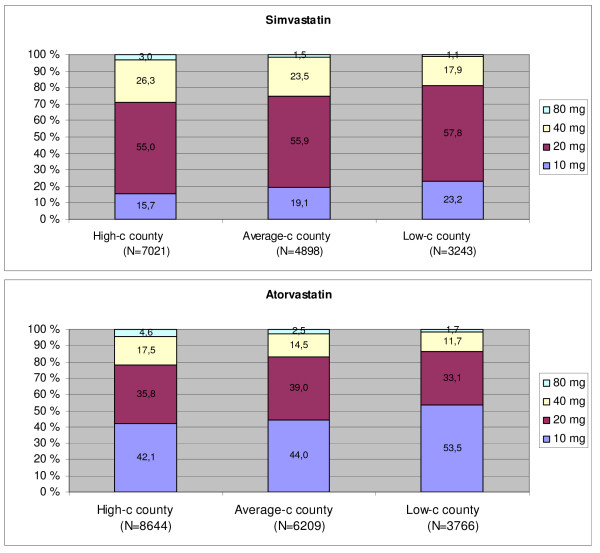
**Proportion of statin users according to tablet strength of simvastatin and atorvastatin in three Norwegian counties ^1^. Norwegian Prescription Database 2004**. High-c county: abbreviation for high-consumption county. ^1^Tablet strength according to the first prescription dispensed in 2004.

More users in the high-consumption county were prescribed simvastatin and atorvastatin in the upper range of the clinically available strengths, including 'high-dose therapy' (atorvastatin and simvastatin of 80 mg) (Figure 4). The high-consumption county had the highest PDD for simvastatin (25.9 mg) and atorvastatin (21.9 mg) (Table 1). The estimated PDDs for all relevant statins were higher than the DDDs – up to twice the DDD for atorvastatin.

**Table 1:** Mean prescribed daily dose (PDD) in milligrams of the different statins in three Norwegian counties^1^. Norwegian Prescription Database 2004

Statin substance (C10AA)	DDD^2^	High-c county (Hedmark) mg	Average-c county (Oppland) mg	Low-c county (Troms) mg
Simvastatin (C10AA01)	15	25.9	24.0	22.5
Lovastatin (C10AA02)	30	31.8	30.3	28.2
Pravastatin (C10AA03)	20	32.1	32.1	32.3
Fluvastatin (C10AA04)	40	60.9	58.7	57.3
Atorvastatin (C10AA05)	10	21.9	19.8	18.0

High-c county: abbreviation for high-consumption county

^1^Mean PDD: calculated for all statins from the total volume dispensed in milligrams divided by the total number of dispensed tablets

^2^The official DDD assigned by the WHO Collaborating Centre for Drug Statistics Methodology, Oslo, Norway [10].

### Continuity of treatment (tablets/day)

The number of prescriptions followed by another prescription in the same individual were 43 045 in 17 954 individuals in Hedmark, 29 971 in 13 348 individuals in Oppland and 19 770 in 8841 individuals in Troms.

The distribution of the mean number of tablets per day for all prescriptions followed by another prescription was similar in the counties. Medians (IQR) in these distributions were 1.05 (0.94–1.39), 1.04 (0.94–1.34) and 1.05 (0.93–1.42) in Hedmark, Oppland and Troms, demonstrating that the users retrieved statins corresponding to the use of one tablet per day.

## Discussion

Although differences in age-distribution seems to be an important variable in the explanation of differences statin consumption, it does not fully outweigh the inter-county variation of statin consumption in Norway. Variations in prevalences of use, and treatment intensity in terms of PDD and choice of statin also affect the total consumption.

Not surprisingly, the estimated PDDs for all statins were higher than the DDDs – up to twice the DDD for atorvastatin. The official DDDs for all statins have remained unchanged since 1994 and have not been adjusted to the new clinical documentation published over the last decade [10]. As indicated from all statin prescriptions dispensed for the first time in 2004; extensive use of atorvastatin, combined with a systematically higher PDD for all relevant statins in the high-consumption county Hedmark, influences total level of, and thereby variation in, statin consumption.

A limitation with this study is the lack of information on statin use in relation to other relevant patient characteristics such as cardiovascular morbidity and risk factors, including clinical measurements. However, the tendency of an overall more aggressive cholesterol treatment in the high-consumption county, Hedmark, is in line with previous findings from our population-based study in Hedmark and the average-consumption county, Oppland: a higher proportion of the statin users in Hedmark achieved the nationally recommended TC target [4]. Although we do not know to what extent LLDs dispensed actually are ingested, patterns of the number of tablets dispensed per day were similar between the counties in our present study. Thus, the success in achieving TC target in Hedmark may most likely be attributed the use of higher dosages, rather than increased continuity of use.

Overall achievement of TC target among statin users in our previous study in Hedmark and Oppland were, however, suboptimal. Only 40–60% of the statin users achieved the recommended TC target, consistent with results from other population-based studies in Norway from the same period [11,12]. Although the presentation of tablet strength according to the first statin prescription dispensed do not take into account a future uptitration of the dose among incident users, it still seems to be a potential for optimising dosing of statins. For example, in our study, only 20–30% of all simvastatin users were on doses corresponding to the dose (40 mg) used in recent pivotal trials, such as the Heart Protection Study [13].

The observed variations in prevalences of statin use in our present study, may be explained by previous findings of a varying threshold for the initiation of statin therapy for primary prevention between the high- and average-consumption counties Hedmark and Oppland. Interestingly, this previous study identified a gap between current practice and guideline recommended level of statin use for primary prevention in both counties [4]. In this situation, the variation in prevalence of statin use today may be small compared with a "scenario" of full implementation of guidelines in either of the regions.

The prevalence patterns of statin use, however, were similar in the counties: increasing with age, peaking in the age group 70–79 years in both sexes. A third of all 70- to 79-year-old men in the high-consumption county, Hedmark, had at least one statin prescription dispensed in 2004. As a comparison, a quarter of all 75-year-old men reported use of a statin in this county in 2000–1 [4]. The age pattern of current statin use seems to be oriented towards more extensive use among elderly people, compared with the situation in the 1990s, where the peak of statin use was observed in individuals aged ten year younger [14-16]. This shift could be explained by several factors:

1. First, statin therapy would normally be a lifelong treatment. Naturally, the age of middle-aged patients, in whom the efficacy was initially documented and therefore recommended in the 1990s, will increase over time. Most patients seems adhere to treatment for many years in Norway [17,18] and other populations with comparable reimbursement systems, such as in Denmark [19,20].

2. Second, recent statin trials have extended our knowledge of the benefit of statin use in elderly people up to about 80 years of age, in secondary prevention of CVD in particular [13,21]. Hence, current Norwegian guidelines now discuss statins for secondary prevention up to this age [22]. The high prevalence in this age group in our study may reflect recommendations being adopted in clinical practice. Corresponding age trends are seen in Denmark: the 75–84 year olds had the highest relative increase in statin use in a study of myocardial infarction (MI) patients in the period 1995–2002 [23].

In our study we found a markedly lower statin use among those aged 80+ compared with the 70–79 year olds in all three regions. These figures are parallel to findings from Norwegian hospitals. The majority of all MI patients aged up to 80 years were discharged with a statin, compared with one in ten of those aged 80 and above [17]. Documentation of beneficial effects of statins in very old people is limited, and our observations may reflect the scepticism among Norwegian doctors to prescribe statins based on extrapolation from evidence among younger individuals.

## Conclusion

The Norwegian Prescription Database offer new opportunities to study different aspects of statin use in individual patients, complementary to information attained from Norwegian health surveys. Differences in age-distribution cannot fully outweigh the variation in statin consumption between counties in Norway. Variations in prevalences of use, and treatment intensity in terms of PDD and choice of statin also affect the total consumption, which correspond to previous findings of more frequent statin use in primary prevention, and more statin users achieving lipid goal in the highest consuming county. The PDDs are higher than the DDDs, up to twice the DDD for atorvastatin. Interpretation of the published increase in statin wholesales over the years, with national and international differences in consumption, should take into account the age-distribution, the differences in the relationship DDD/PDD and the choice of statin.

## Authors' contributions

IH, Ssa, AE, KF and SSk have made contributions to acquisition and analysis of the prescription data. All authors have been involved in the interpretation of the data, drafting and revising the manuscript, and approval of the final manuscript.

## Pre-publication history

The pre-publication history for this paper can be accessed here:


